# Predicting unplanned readmissions in neurological patients: A large-scale AI-driven analysis using CHAID decision trees

**DOI:** 10.1371/journal.pone.0360077

**Published:** 2026-10-09

**Authors:** Randa Al-Kharabsheh, Muayyad Ahmad

**Affiliations:** 1 Critical Care Nursing, Clinical Nursing Department, School of Nursing, University of Jordan, Amman, Jordan; 2 Department of Training and Continuing Education, King Hussein Medical Hospital, Jordanian Royal Medical Services/Army Forces, Amman, Jordan; 3 Clinical Nursing Department, School of Nursing, University of Jordan, Amman, Jordan; Harvard Medical School, UNITED STATES OF AMERICA

## Abstract

**Background:**

Unplanned readmissions in neurology signify care gaps, increasing costs, and morbidity. This study aimed to develop and internally validate an AI model to predict unplanned readmissions among neurological patients in Jordan.

**Method and materials:**

We conducted a retrospective analysis of 2,795 adult neurology admissions (2022–2024). Patients’ diagnosis was grouped and analyzed based on the International Classification of Diseases and Related Health Problems, Tenth Revision. Nine prediction models were compared using Electronic Health Record (EHR) data. The Chi-square Automatic Interaction Detection (CHAID) decision tree was selected for optimal performance and interpretability.

**Results:**

The unplanned readmission rate was 8.9%, highest in the neoplasm group (24%). The CHAID model demonstrated modest discriminative ability (AUC = 0.671). For risk stratification, it achieved a balanced accuracy of 0.63, sensitivity of 0.52, and specificity of 0.74. Key predictors were Charlson Comorbidity Index (CCI ≥ 5), age, geographic region, length of stay, and sex. Risk-stratification metrics were calculated descriptively from the complete cohort, whereas independent model validation was based on the held-out test-set AUC. Subgroup analysis revealed distinct risk profiles: vascular patients with high CCI (p < .001, χ2 = 66.368), young females of whom 37.3% had neoplasms (p = .037, χ2 = 4.367), and functional disorder patients from northern regions (p = .009, χ2 = 8.897).

**Conclusion:**

This is one of the first studies from the Middle East to demonstrate the potential of the CHAID model for risk stratification in neurology readmissions. The model offers an interpretable framework to identify high-risk patient subgroups but requires external validation before clinical application. Its poor-to-fair discriminative performance (AUC = 0.671) supports population-level risk stratification rather than individual-level prediction.

## Introduction

Unplanned readmissions, defined as a return to the hospital within 30 days of discharge from general care or 90 days for critical care for reasons related to the index admission, are often linked to urgent health crises and lead to increased morbidity, mortality, and financial costs [1,2]. The most common window for unplanned readmissions that aims for quality improvement and benchmarking between health institutions was 30 days for both floors and critical care units. However, longer window had been reported in the literatures using the 90-day and the 1-year windows for critical care patients as a strong indicator for longer-term prognosis especially for complex populations such as neurological patients who needs longer recovery, delayed complications and declined functional abilities [3]. We used in this study the 90-day window to document unplanned readmissions for neurological patients had an index admission into critical care units.

These readmissions represent a significant challenge in modern healthcare, signaling potential gaps in care continuity and imposing substantial burdens on both patients and healthcare systems [4]. In the specialized fields of neurology and neurosurgery, where patients often have complex, chronic conditions, identifying those at high risk for readmission is crucial. Targeted interventions, such as enhanced discharge planning, patient education, and structured outpatient follow-up, hinge on the ability to accurately predict which patients are most vulnerable [5]. Therefore, developing reliable, clinically interpretable prediction models is a critical step toward improving patient outcomes and optimizing resource allocation.

Globally, unplanned readmissions following neurological care are a well-documented concern, with studies highlighting variable rates and associated risk factors. For instance, research in the United States has reported readmission rates ranging from 5.8% to over 20%, depending on the specific neurological diagnosis, with conditions like brain tumors and stroke carrying particularly high risks [6,7]. Factors such as advanced age, high comorbidity burden (measured by indices like the Charlson Comorbidity Index), longer hospital stays, and specific postoperative complications are consistently identified as key predictors [6,8,9]. These findings underscore a universal need for effective risk stratification tools to guide proactive care transition strategies.

However, this evidence base has a significant geographical gap. In the Middle East, and specifically in Jordan, there is a notable lack of large-scale, data-driven studies investigating the rates and predictors of unplanned readmissions among neurological and neurosurgical patients. The Jordanian healthcare context, with its unique demographic profile, healthcare delivery structure, and disease patterns, may present distinct risk factors and challenges. This gap limits the ability of local clinicians and administrators to develop evidence-based, tailored interventions to reduce readmissions, potentially leading to preventable patient hardship and inefficient use of limited healthcare resources.

To address this gap, this study had three primary purposes: (1) to determine the incidence of unplanned readmissions among neurological patients in Jordan's largest health sector; (2) to identify the key demographic, clinical, and geographic predictors of these readmissions; and (3) to develop and internally validate a clinically interpretable artificial intelligence (AI) prediction model. Utilizing a large-scale dataset from national Electronic Health Records (EHRs), we employed and compared several AI models, ultimately selecting the Chi-square Automatic Interaction Detection (CHAID) decision tree for its optimal balance of predictive accuracy and transparency. The novelty of this work lies in its application of AI-driven segmentation to uncover subgroup-specific risk profiles within a Middle Eastern population, thereby providing an actionable framework for personalized discharge planning and transitional care designed for the local context.

## Methods

### Study design and data source

A retrospective analysis was conducted on data from 2,795 adult patients admitted for neurological/neurosurgical reasons between November 2022 and August 2024. Patients’ diagnosis was categorized and analyzed based on the International Classification of Diseases and Related Health Problems (ICD), Tenth Revision. The patients were categorized into neoplasm, vascular and functional groups. The TRIPOD Checklist: Prediction Model Development was followed as a reporting guidance in this study.

Data were extracted in October 2025 from the national Electronic Health Solutions (EHS) database, managed anonymously by Health Data Analytics (HDA) and provided in separate parts for the authors with unidentifiable patient IDs. Subsequently, the authors had no access to patients’ records.

### Variables and data preparation

The unplanned readmissions were not distinguished from planned readmissions at EHS system. They provided us with the admission dates and causes of admissions. We tested all the admissions and then categorized them into planned and non-planned according to the unplanned readmission definition. The period calculated from the readmission date and the index discharge date; all those who were readmitted in a period of less than 30 days for floor index admissions and less than 90 days for critical care index admissions were candidates for unplanned readmission group. Then, the causes for all candidate readmission were tested; if these readmissions had any attribute that related to the index cause categorized as unplanned readmissions, all other admissions categorized as planned readmissions. Because of fragmented and inconsistent data provided by EHS system, we used only the data for the last two admissions for those patients who had multiple readmissions.

Data included the following independent variables: 1) demographics, 2) primary diagnosis (categorized into Neoplasm, Vascular, and Functional groups), 3) comorbidities; the EHS system had used the ICD coding and classification system to categorize all the comorbidities. The ICD Ninth Revision was used till August 2017 then the system updated into the ICD Tenth Revision. The EHS system provided all documented comorbidities for all patient involved in the study over a lookback period started from February 2015 till August 2024. All those comorbidities involved in the Charlson Comorbidity Index (CCI) calculation were involved in the analysis in addition to the calculated CCI scores, 4) geographic location (governorate); divided into northern, southern, and middle governorate. However, the CHAID model manipulated and merged governorate categories and ended with two subgroups that share same characteristics in relation to the dependent variable; the northern one versus the southern/middle one, 5) length of stay (LOS); which is the period from the admission and discharge dates for the same hospitalization, 6) admission site (critical care/general ward), and 7) key laboratory values; we used only the first result for each laboratory test at the index admissions. Data cleaning, merging, and transformation were performed using the Statistical Package for the Social Sciences (IBM SPSS) Statistics 27. We represent in Fig 1 the data extraction, processing, and analysis procedures.

**Fig 1:**
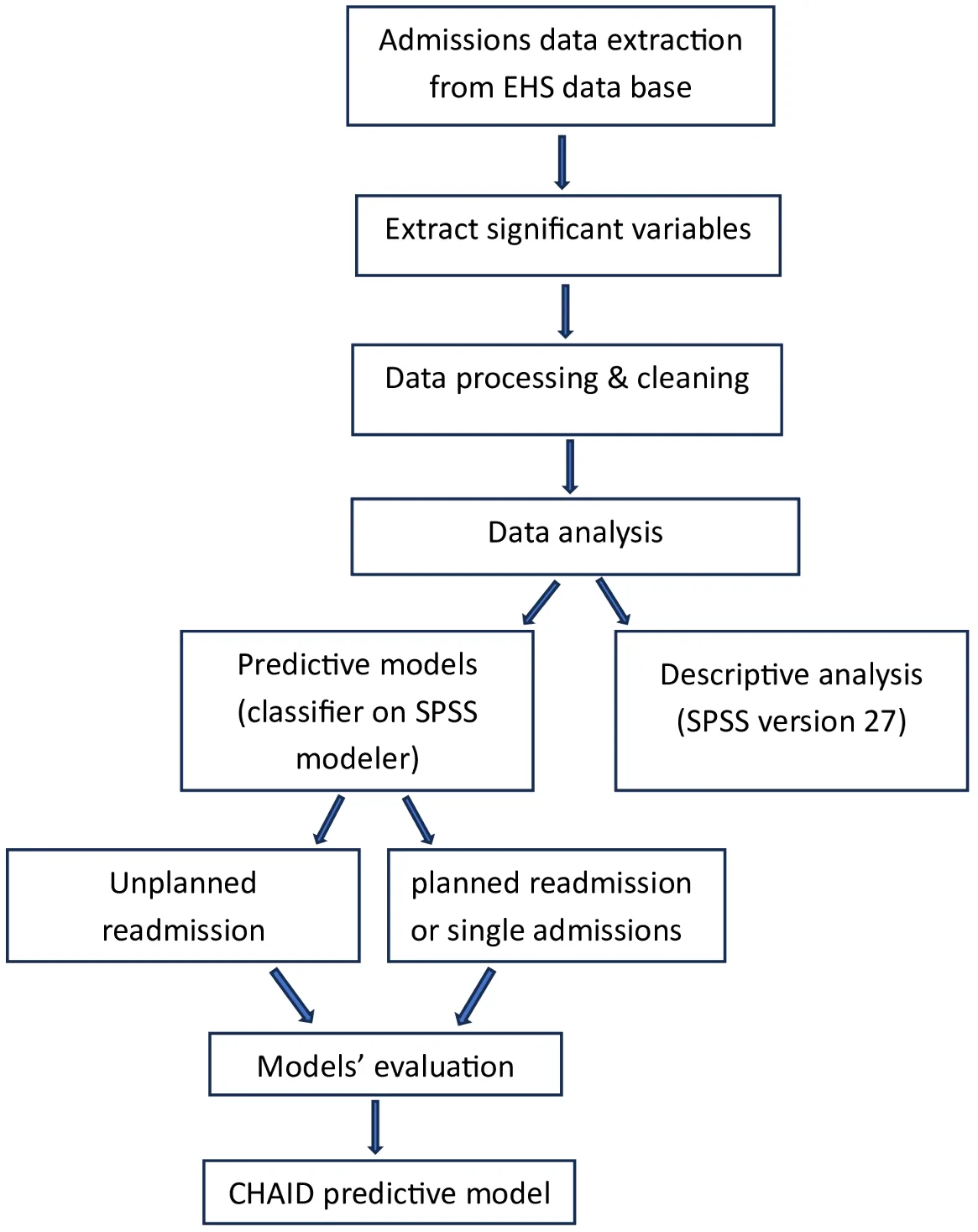
Data extraction, processing, and analysis procedures.

### Data processing and cleaning

We analyzed the data for noise, missing values, and inconsistency using outlier detection and frequency description in the IBM-SPSS Program (version 27.0). We excluded from the analysis all duplicated redundant data, and all variables that were either not provided by the EHS database or provided with huge missing or/and illogical readings. As an example of excluded variables, height, weight, symptom data, physician and nursing notes, and certain laboratory tests (such as lactate dehydrogenase, high-density lipoprotein (HDL), albumin, cholesterol, and fasting blood sugar data).

### Missing data assessment

Of the original 54 candidate variables, 12 were excluded a priori due to >20% missingness: height (41%), weight (38%), lactate dehydrogenase (52%), HDL (44%), albumin (35%), cholesterol (47%), fasting blood sugar (39%), and five clinical note fields (65 89%). For the remaining 42 variables, missingness was < 3% per variable; thus, complete case analysis was applied (final n = 2,795). No imputation was performed because missingness in retained variables was very low, and excluded variables had excessively high missing rates where imputation would be unreliable. This exclusion may introduce bias if missingness is not completely at random, which we now note as a limitation.

### Predictive modeling and selection

The SPSS Modeler 18 was used to build and compare nine prediction models (e.g., CHAID, C5.0, Logistic Regression, Neural Networks). Model performance was evaluated based on overall accuracy and the Area Under the Curve (AUC). The CHAID model was selected as the final model due to its superior balance of accuracy (91.1%) and interpretability (AUC = 0.671). The CHAID is a decision tree algorithm that uses chi-square tests to identify the most significant predictor for splitting the data at each node. It handles categorical variables effectively and produces an intuitive tree structure. Hyperparameters were set to a maximum tree depth of three levels, with a statistical significance (p < 0.05) threshold for splitting. A train-test split method was used for validation.

### Model development and validation

The dataset was randomly split into training (70%, n = 1,956) and test (30%, n = 839) sets using SPSS Modeler’s random number generator with a fixed seed (12345) to ensure reproducibility. No stratification by outcome was applied; the split preserved the overall readmission rate (training: 8.8%, test: 9.0%). Internal validation was performed on the held-out test set only; no cross validation was used. All preprocessing parameters (e.g., one hot encoding categories, z score means/SDs) were derived from the training set only and then applied to the test set to prevent data leakage.

### Preprocessing and missing data

Categorical variables (sex, region, admission site, diagnosis group) were one hot encoded. Continuous variables (age, LOS, laboratory values) were standardized (z score) only for Neural Net 1 and Discriminant 1; tree-based models and logistic regression used raw values. Variables with >20% missing were excluded a priori (height, weight, lactate dehydrogenase, HDL, albumin, cholesterol, fasting blood sugar). For retained variables, complete case analysis was used (missingness <3%).

### Hyperparameter settings (all nine models)

All nine models were trained using default IBM SPSS Modeler 18 parameters except where noted. CHAID used a maximum depth of 3, minimum parent size of 100, minimum child size of 50, α split = 0.05, α merge = 0.05, and chi square splitting. C5.1 used pruning severity of 75% and minimum cases per child = 2. Quest used α split = 0.05, pruning enabled, and minimum parent size = 50. C&R Tree 1 used max depth = 5, min parent size = 50, and min child size = 25. Neural Net 1 used an automatic one hidden layer, max training time of 15 minutes, and tolerance = 0.0001. Logistic Regression 1 used stepwise entry (p ≤ 0.05) and removal (p ≥ 0.10). Bayesian Network 1 used hill climbing, max parents = 3, and BIC scoring. Discriminant 1 used stepwise Wilks’ lambda with entry F = 3.84 and removal F = 2.71. Decision List used confidence = 0.9, min support = 2%, and max rules = 50. No hyperparameter optimization (grid search / cross validation) was performed.

### Model comparison

Nine models were compared on the test set (S1 Table). The CHAID was selected because it demonstrated the highest discriminative ability among the interpretable tree-based models (AUC = 0.671) while maintaining a transparent decision-tree structure. Although several models achieved the same overall accuracy (91.1%), three models (C5.1, Quest, and C&R Tree 1) had AUC = 0.5 (no better than random), confirming that accuracy alone was uninformative in this imbalanced dataset.

### Ethical considerations

Ethical approval was obtained from the Jordanian Royal Medical Services (Document No. T F-3/1–6626, Meeting No. 9/2024). Patient consent was waived as the analysis used fully anonymized data; this eliminates some risks for selection bias.

## Results

### Sample characteristics and readmission rates

Among 2,795 patients, 249 (8.9%) experienced an unplanned readmission. As presented in S2 Table, among those who had floor index admissions; a total of 211 (8.5%) patients experienced 30-day unplanned readmissions, while among those who had critical care index admissions; a total of 38 (11.8%) patients experienced 90-day unplanned readmissions. The neoplasm group reported the higher incidence of unplanned readmissions (24%); the higher 30-day floor unplanned readmissions (22.7%) and 90-day critical care admissions (50%). Among those who had index admission into critical care units, 11.4% in the vascular group and 5.2% in the functional group experienced 90-day critical care unplanned readmissions. Returning into critical care units among the unplanned readmissions differed between the study groups. In the neoplasm group, five patients (9.6%) out of the 52 patients who had unplanned readmissions were returned into critical care units. Meanwhile, 5 patients (4.6%) out of 109 patients in the vascular group and three patients (3.4%) out of the 88 patients in the functional groups had returned to critical care units in the unplanned readmissions.

S3 Table shows that the diagnosis-related complications (e.g., tumor progression, disc issues) were the most frequent cause of unplanned readmission overall (45.8%). Among the vascular group, which reported the higher critical care unplanned readmission, recurrent brain hemorrhage followed by cerebrovascular accident (CVA) reported the higher causes of unplanned readmissions (29.4% and 18.3%). Spinal disc disease reported the most common cause of unplanned readmissions (59.1%) among the functional group, which had the higher floor unplanned readmissions.

### Key predictors from bivariate analysis

The overall mean age was 56.1 years (SD = 17.6), and 59.5% were male. As shown in S4 Table, the unplanned readmission rate was higher for index critical care admissions (11.8%) compared to general ward admissions (8.5%). We used independent sample T test for continuous variables, while the chi-square test was used for all the categorical variables. The analysis yielded significant bivariate predictors of unplanned readmission for the entire cohort included a CCI score ≥3 (p = 0.004), index admission to a critical care unit (p = 0.05), hyponatremia (p = 0.04), and history of cerebrovascular disease (p = 0.003), diabetes (p = 0.01), and renal disease (p = 0.04). Group-specific analyses found that in the vascular group, a high CCI (p = 0.01), history of cerebrovascular disease (p < 0.001), ischemic heart disease (p = 0.05), and critical care admission (p = 0.02) were significant. In the functional group, older age (p = 0.03), diabetes (p = 0.01), and abnormal lab values (white blood cells (WBC) [p = 0.03], platelets[p = 0.02]) were significant. Meanwhile, only the length of stay was significant in the neoplasm group (p = 0.03).

### The CHAID prediction model

Although the bivariate analysis used a prespecified CCI threshold (≥3) for univariable comparison, the CHAID algorithm treated CCI as a predictor and automatically identified the split points that best separated patients according to readmission risk. Consequently, the final CHAID tree produced different CCI groupings (0, 1–4, 5–6, and 7), with the highest-risk subgroup emerging at CCI ≥ 5.

As presented in Fig 2, the CHAID decision tree identified CCI as the primary (root) predictor (Chi-square = 66.37, p < 0.001), splitting patients into four nodes. Due to class imbalance (8.9% readmission rate), we report additional metrics. Following selection of the final CHAID model using the independent train-test comparison, the final decision tree was applied descriptively to the complete cohort (N = 2,795) to characterize clinically interpretable patient subgroups. Consequently, the confusion matrix and the derived sensitivity, specificity, precision, F1 score, and balanced accuracy reported below were calculated from the complete cohort and are intended to summarize the subgroup-classification characteristics of the final tree rather than to provide independent out-of-sample validation. Independent model validation is represented by the held-out test-set AUC (0.671). The model revealed distinct pathways:

1. **High CCI (Scores 5–7; n = 365):** Patients, predominantly with vascular diagnoses (CVA, hemorrhage) as presented in S5 Table, formed the highest-risk groups (readmission rates: 15.3% [n = 51] for CCI 5–6, 38.7% [n = 12] for CCI 7). No further significant predictors were found for this high-risk cohort.
2. **Medium CCI (Scores 1–4; n = 1,751):** For this node, age was the secondary predictor.
  - **Age < 40 (n = 166):** Sex was a third predictor, with females having a higher readmission rate (30.4%; n = 17). Neoplasm was found among 37.3% of these young females.
  - Age 40–79 (n = 1,513): Among this aging subgroup, vascular problems were the dominant mainly CVA (32.1%; n = 506) and brain hemorrhage (28.5%; n = 452). Length of stay (LOS) was a third predictor among these patients with LOS > 13 days had a higher readmission rate (13.8%; n = 20).
  - **Age ≥ 80 (n = 72):** This group had a very low readmission rate (1.4%; n = 1); presented primarily with brain haemorrhage (68.1%; n = 49).
3. **CCI = 0 (n = 679):** Geographic region was the secondary predictor. The CHAID model divided the geographic region into two main subgroups; the northern one (n = 110) and the middle/south regions (n = 569). Patients from the northern governorate had a significantly higher readmission rate (10%; n = 11) compared to those from the middle/south (3.5%; n = 20). This group consisted almost entirely of functional disorder patients (89.4%; n = 607), mainly spinal disc disease (72.5%; n = 492).

**Fig 2:**
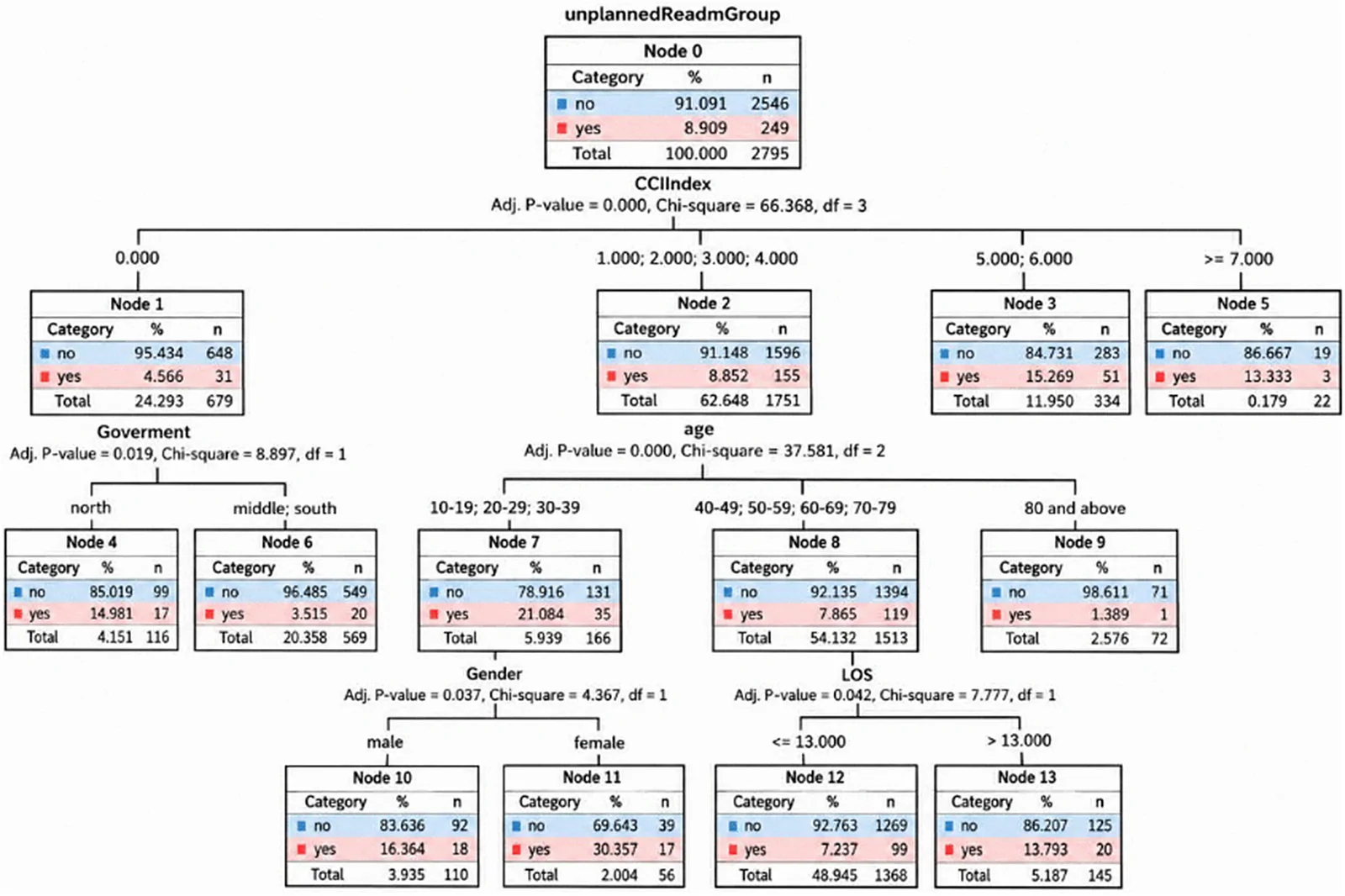
Final CHAID decision tree generated from the complete study cohort (N = 2,795) for descriptive characterization of clinically interpretable patient subgroups. Independent model validation was performed separately using the held-out test set (n = 839).

## Discussion

This study presents one of the early studies that used an AI-driven, large-scale analysis of unplanned readmissions in a Middle Eastern neurological cohort, yielding critical insights with both regional and global relevance. Our overall unplanned readmission rate of 8.9% and both the specific 30-day floors and 90-days critical care units’ rates (8.5% and 11.8%) aligned with the lower range reported internationally for neurosurgical populations, which often vary from 9% to over 24% depending on case mix and healthcare systems [6,7,10–12]. The 90-day unplanned readmissions rates for cases that had index admission in critical care provides quality measures to improve the discharge decision from critical care units and enhance the transition care as these patients had higher mortality rates and more complex comorbidities [13].

The elevated risk observed within our neoplasm group (overall rate = 24%; floors rate = 22.7% and critical care rate = 50%) resonates strongly with global literature, where brain tumor patients consistently demonstrate some of the highest readmission rates, primarily due to disease progression and treatment-related complications [6,7]. However, our findings challenge some international patterns; while Western studies often identify vascular diagnoses as the highest-risk category, our cohort’s vascular group had a lower rate (7.7%), potentially reflecting differences in patient acuity, access to acute stroke care, or follow-up structures in our setting [10]. This divergence underscores the necessity of developing context-specific models rather than relying solely on imported risk algorithms.

The core contribution of this research lies not merely in identifying predictors, but in using the CHAID model to reveal how these predictors interact to define distinct, clinically intuitive patient archetypes. These archetypes should be interpreted as descriptive patient profiles derived from the final CHAID tree rather than independently validated prediction rules. Their principal value lies in generating clinically meaningful hypotheses and supporting quality-improvement initiatives, whereas predictive performance was assessed separately using the held-out test set. This moves beyond traditional logistic regression outputs to provide a decision-tree logic that clinicians can visualize and apply [14]. The algorithm identified three high-risk pathways: the high-comorbidity vascular patient (CCI ≥ 5), the young female of whom 37.3% had neoplasms (CCI 1–4, age < 40), and the low-comorbidity functional patient from a peripheral region. This stratification offers a nuanced understanding that a simple “high-risk” label cannot. For example, the finding that young females with brain tumors are a particularly vulnerable subgroup adds specificity to the literature, which often highlights age or comorbidity but less frequently identifies this interaction with sex in non-malignant tumor management.

Our model’s selection of the CCI as the primary split variable reinforces its established role as a powerful prognostic tool across medicine, validating its utility in neurosurgical outcomes within our population. The subsequent branching into age, region, and length of stay constructs a logical clinical narrative. The association between prolonged length of stay and increased readmission risk in vascular patients mirrors findings in stroke cohorts, suggesting index hospitalization complexity as a proxy for fragility [9,15]. Conversely, the geographic disparity observed in the functional group—where patients in northern governorates had triple the readmission risk—is a novel and significant finding. This is likely indicative of systemic issues of healthcare access, where patients with manageable conditions like disc disease may rely on episodic inpatient care due to limited outpatient rehabilitation or pain management services in their regions, a factor less commonly highlighted in studies from systems with more uniform resource distribution. Karanjia, Dugyala [16] highlights the importance of updating and standardizing health care in addition to resources allocation for this specific populations, which is an essential element in decreasing unplanned readmissions and its associated costs.

We deliberately selected the CHAID model over others, potentially more complex “black-box” algorithms like neural networks due to its paramount advantage: interpretability [17]. In clinical practice, a model’s utility is determined not only by its statistical performance but by its ability to be understood and trusted by clinicians. The CHAID tree functions as a visual clinical decision aid, allowing a provider to trace a patient’s path through the nodes based on their characteristics [18].

This transparency could, in principle, facilitate future integration into clinical workflows, but such integration would first require prospective validation and demonstration of clinical utility – which are beyond the scope of this study. The AUC of 0.671 indicates poor-to-fair discrimination and considerable room for improvement, although it falls within the range reported for several published neurology readmission models (approximately 0.60–0.73). However, this performance level is insufficient for individual-level prediction or for deploying the model as a real-time clinical decision aid. Therefore, we do not recommend clinical implementation based on the current results. Instead, the model should be viewed as a hypothesis-generating tool for quality improvement and research, identifying patient subgroups that warrant further study and targeted intervention. Prospective external validation in independent Jordanian cohorts is required before any clinical use can be considered.

### Clinical utility and external validation needs

We acknowledge that an AUC of 0.671 represents poor-to-fair discriminative ability, limiting the model's suitability for individual level prediction while still supporting exploratory population level risk stratification. This performance is comparable to existing readmission prediction models in neurology, where reported AUCs typically range from 0.60 to 0.73. For example, Sander et al. (2020) reported AUCs of 0.62–0.68 for neurosurgical readmissions, and Hu et al. (2025) achieved an AUC of 0.73 in stroke patients with diabetes. Although the overall model comparison was performed using an independent train-test split, the confusion matrix, sensitivity, specificity, precision, F1 score, and balanced accuracy were calculated descriptively from the complete cohort after development of the final CHAID tree. Consequently, these statistics should not be interpreted as independent validation metrics and are likely to be optimistically biased. Future studies should evaluate all classification metrics using external validation cohorts or independent test datasets. Assessing generalizability, external validation in independent Jordanian healthcare sectors (e.g., university hospitals, private facilities) is necessary before clinical implementation. Future work should also evaluate the model's impact on discharge planning and readmission rates through prospective interventional studies.

### Limitations related to model performance and methodology

Several limitations should be considered. First, the low incidence of unplanned readmissions (8.9%) created a class imbalanced dataset; overall accuracy (91.1%) overstates performance, whereas the corrected full-cohort balanced accuracy (0.63) provides a more realistic estimate of risk-stratification performance. Three comparator models achieved the same accuracy but had AUC = 0.5 (no better than chance), underscoring that accuracy alone is inappropriate for imbalanced outcomes. Second, we used a simple 70/30 train test split without cross validation or temporal validation. Third, hyperparameters were prespecified (or left at defaults) rather than optimized, which may have underestimated some models’ potential. Fourth, complete case analysis excluded <3% of records but could still introduce bias. Fifth, the random split was not stratified by outcome, though training and test readmission rates were nearly identical. Sixth, the poor-to-fair discriminative performance (AUC = 0.671) and absence of external validation mean this model is not suitable for clinical implementation at present; it should be used only for hypothesis generation and risk stratification in research or quality improvement contexts. Future work should employ k fold cross validation, repeated resampling, synthetic oversampling, cost sensitive learning, time based splitting, and hyperparameter tuning to improve model sensitivity and generalizability.

In conclusion, this study provides an interpretable framework for population-level risk stratification of neurological readmissions rather than a highly accurate individual prediction model. Although the CHAID model demonstrated poor-to-fair discrimination (AUC = 0.671; balanced accuracy = 0.63), it successfully identified clinically meaningful high-risk patient subgroups that may support quality-improvement initiatives after external validation. The resulting patient profiles offer a hypothesis generating blueprint for targeted quality improvement efforts (e.g., enhancing transitional care for high comorbidity vascular patients, supporting young neuro oncology patients, and addressing regional access gaps for spinal care). However, the model’s poor-to-fair discriminative performance and lack of external validation indicate that it should currently be regarded as a research and quality-improvement tool rather than a clinically deployable prediction model.

## Supporting information

S1 Table Performance of nine candidate prediction models. (DOCX)

S2 Table Unplanned Readmission Rates among Diagnosis Groups at the Index Admission. (DOCX)

S3 Table Causes of Unplanned Readmissions for Patients with Neurological Conditions. (DOCX)

S4 Table Bivariate Analysis for Patients’ Characteristics at Index Admission for the cohort study groups. (DOCX)

S5 Table Causes of Index Admissions within the sub-Categories of Predictors on CHAID^ Model. ^^^CHAID: Chi-square Automatic Interaction Detection.
(DOCX)
