# Supplementary material for: Predicting unplanned readmissions in neurological patients: A large-scale AI-driven analysis using CHAID decision trees
Source: PLoS One. 2026 Oct 9;21(10):e0360077. doi: 10.1371/journal.pone.0360077 (PMC13652259; doi:10.1371/journal.pone.0360077)
Supplement: Supplementary file 1 [file pone.0360077.s001.docx]

| **Table S1: Performance of nine candidate prediction models (test set, n=839)** | | |
| --- | --- | --- |
| Model | Overall Accuracy (%) | Area Under Curve (AUC) |
| CHAID* | 91.091 | 0.671 |
| C5.1 | 91.091 | 0.5 |
| Quest 1 | 91.091 | 0.5 |
| C&R Tree 1 | 91.091 | 0.5 |
| Bayesian Network 1 | 68.837 | 0.658 |
| Logistic Regression 1 | 68.587 | 0.612 |
| Decision List 1 | 84.615 | 0.539 |
| Discriminant 1 | 66.082 | 0.531 |
| Neural Net 1 | 68.658 | 0.509 |
| *CHAID: The Chi-square Automatic Interaction Detection | | |
