# Supplementary material for: Predicting unplanned readmissions in neurological patients: A large-scale AI-driven analysis using CHAID decision trees
Source: PLoS One. 2026 Oct 9;21(10):e0360077. doi: 10.1371/journal.pone.0360077 (PMC13652259; doi:10.1371/journal.pone.0360077)
Supplement: Supplementary file 2 [file pone.0360077.s002.docx]

| **Table S2: Unplanned Readmission Rates among Diagnosis Groups at the Index Admission (N=2,795)** | | | | | | |
| --- | --- | --- | --- | --- | --- | --- |
| Cause of index admission | Frequency  n (%) | ICU admissions  321 (11.5%) | Unplanned readmission  249 (8.9%) | 30-day Floor Unplanned readmission  211 (8.5%) | 90-day ICU Unplanned readmission  38 (11.8%) | Returning into ICU  13 (5.2%) |
| Neoplasm group: |  |  |  |  |  |  |
| Brain tumor | 217 (7.8) | 10 (4.6) | 52 (24) | 47 (22.7) | 5 (50) | 5 (9.6) |
| Vascular group: | 1409 (50.4) | 273 (19.4) | 109 (7.7) |  |  |  |
| CVA^+TIA^^ | 760 (27.2) | 63 (19.6) | 51 (20.5) | 78 (6.9) | 31 (11.4) | 5 (4.6) |
| AVM^^^ | 21 (0.8) | 2 (0.6) | 2 (0.8) |  |  |  |
| Brain hemorrhage | 628 (22.38) | 208 (64.8) | 56 (22.5) |  |  |  |
| Functional group: | 1169 (41.8) | 38 (3.3) | 88 (7.5) | 86 (7.6) | 2 (5.2) | 3 (3.4) |
| Spinal Disc | 914 (32.7) | 1 (2.6) | 70 (28.1) |  |  |  |
| Back pain | 28 (1) |  | 3 (1.2) |  |  |  |
| Low LOC^^^^ | 69 (2.5) | 23 (60.5) | 3 (1.2) |  |  |  |
| Seizure/ epilepsy | 120 (4.3) | 13 (4) | 10 (4) |  |  |  |
| dizziness | 34 (1.24) | 1 (0.3) | 1 (0.4) |  |  |  |
| High ICP*/ CSF** leak | 4 (0.1%) |  | 1 (0.4) |  |  |  |

^CVA: cerebrovascular accident, ^^ TIA: transient ischemic attack, ^^AVM: arteriovenous malformation, ^^^^ LOC: low level of consciousness, * ICP: intracranial pressure, **CSF: cerebrospinal fluid
