# Supplementary material for: Predicting unplanned readmissions in neurological patients: A large-scale AI-driven analysis using CHAID decision trees
Source: PLoS One. 2026 Oct 9;21(10):e0360077. doi: 10.1371/journal.pone.0360077 (PMC13652259; doi:10.1371/journal.pone.0360077)
Supplement: Supplementary file 3 [file pone.0360077.s003.docx]

| **Table S3: Causes of Unplanned Readmissions for Patients with Neurological Conditions (n=249)** | | | | | |
| --- | --- | --- | --- | --- | --- |
| Cause category | Readmission cause | N (%) | Neoplasm group  (n=52) | Vascular group (n=109) | Functional group (n=88) |
| surgical complications | Brain hemorrhage | 36 (14.5) | 2 (3.8) | 32 (29.4) | 1 (1.1) |
|  | SSI^ | 20 (8.0) | 2 (3.8) | 2 (1.8) | 14 (15.9) |
|  | Fever | 1 (0.4) | ---- | 1 (0.9) | ---- |
|  | High ICP^^ or CSF^^^ leak | 3 (1.2) | ---- | ---- | 3 (3.4) |
|  | **Total** | **60 (24.1)** | **4 (7.7)** | **35 (32.1)** | **18 (20.5)** |
| medical complications | AF^^^^ | 1 (0.4) | ---- | 1 (0.9) | ---- |
|  | Hypoxia | 1 (0.4) | ---- | ---- | 1 (1.1) |
|  | High kidney functions | 11 (4.4) | 1 (1.9) | 9 (8.3) | 1 (1.1) |
|  | Hypotension | 3 (1.2) | ---- | 2 (1.8) | 1 (1.1) |
|  | Edema | 1 (0.4) | ---- | 2 (1.8) | 2 (2.3) |
|  | Hypoglycemia | 1 (0.4) | ---- | ---- | 1 (1.1) |
|  | Hyponatremia | 1 (0.4) | ---- | 1 (0.9) | ---- |
|  | Acidosis | 1 (0.4) | ---- | ---- | 1 (1.1) |
|  | UTI^^^^^ | 7 (2.8) | ---- | 7 (0.8) | ---- |
|  | Sepsis | 3 (1.2) | ---- | 3 (2.8) | ---- |
|  | General weakness | 7 (2.8) | ---- | 7 (6.4) | ---- |
|  | Thrombosis-spleen | 1 (0.4) | ---- | ---- | ---- |
|  | **Total** | **31 (12.4)** | **1 (1.9)** | **32 (29.4)** | 7 (8.0) |
| diagnosis-related complications | AVM* | 3 (1.2) | ---- | 3 (2.8) | ---- |
|  | Angiogram | 3 (1.2) | ---- | 3 (2.8) | ---- |
|  | Spinal Disc | 52 (20.9) | ---- | ---- | 52 (59.1) |
|  | Back pain | 1 (0.4) | ---- | ---- | 1 (1.1) |
|  | Brain tumor or craniotomy | 40 (16.1) | 40 (76.9) | ---- | ---- |
|  | Breast mass | 2 (0.8) | 2 (3.8) | ---- | ---- |
|  | Lymph node enlargement | 1 (0.4) | ---- | ---- | 1 (1.1) |
|  | Pressure injury | 3 (1.2) | ---- | 1 (0.9) | 2 (2.3) |
|  | DVT** | 5 (2) | 1 (1.9) | 3 (2.8) | 1 (1.1) |
|  | Radiotherapy | 2 (0.8) | 2 (3.8) | ---- | ---- |
|  | Emergency visit | 2 (0.8) | ---- | 2 (1.8) | ---- |
|  | **Total** | **114 (45.8)** | **45 (86.5)** | **12 (11)** | **57 (64.8)** |
| neurological decompensation | CVA*** | 19 (7.6) | ---- | 20 (18.3) | ---- |
|  | Carotid stenosis | 2 (0.8) | ---- | 2 (1.8) | ---- |
|  | Low LOC**** | 6 (2.4) | 1 (1.9) | 4 (3.7) | 1 (1.1) |
|  | Seizure or epilepsy | 10 (4) | 1 (1.9) | 4 (3.7) | 5 (5.7) |
|  | **Total** | **37 (14.8)** | **2 (3.8)** | **30 (27.5)** | **6 (6.8)** |

^SSI: surgical site infection; ^^ICP: intracranial pressure; ^^^CSF: cerebrospinal fluid; ^^^^AF: atrial fibrillation; ^^^^^UTI: urinary tract infection; *AVM: arteriovenous malformation; ** DVT: deep vein thrombosis; ***CVA: cerebrovascular accident; **** LOC: low level of consciousness
