# Supplementary material for: Predicting unplanned readmissions in neurological patients: A large-scale AI-driven analysis using CHAID decision trees
Source: PLoS One. 2026 Oct 9;21(10):e0360077. doi: 10.1371/journal.pone.0360077 (PMC13652259; doi:10.1371/journal.pone.0360077)
Supplement: Supplementary file 4 [file pone.0360077.s004.docx]

| **Table S4: Bivariate Analysis for Patients’ Characteristics at Index Admission for the cohort study groups (N=2,795)** | | | | | | | | | |
| --- | --- | --- | --- | --- | --- | --- | --- | --- | --- |
| Patient characteristics | Entire cohort (N=2795) | | | Neoplasm group (n=217) | | Vascular group (n=1409) | | Functional group (n=1169) | |
|  | Overall  (n=2795) | unplanned readmission  n (%) | Statistics  (p value) | unplanned readmission  n (%) | Statistics  (p value) | unplanned readmission  n (%) | Statistics  (p value) | unplanned readmission  n (%) | Statistics  (p value) |
| Age; mean (SD) | 56.1 (17.6) | 57.7 (18.1) | 0.300 | 52 (16.9) | 0.270 | 66 (17.9) | 0.210 | 51 (14.8) | 0.030 |
| LOS^ mean (SD) | 6.8 (21) | 6.8 (6.8) | 0.700 | 8 (6.1) | 0.030 | 4.8 (4.4) | 0.810 | 8 (6.8) | 0.310 |
| Sex; male n (%) | 1664 (59.5) | 137 (55) | 0.130 | 25 (21.9) | 0.530 | 60 (7.2) | 0.360 | 52 (7.3) | 0.730 |
| CCI^^ (score 3 and above) n (%) | 1144 (40.9) | 124 (49.8) | 0.004 | 31 (25.2) | 0.750 | 80 (9.2) | 0.010 | 31 (59.6) | 0.750 |
| Comorbidities n (%): |  |  |  |  |  |  |  |  |  |
| Cerebrovascular disease | 1062 (38) | 117 (11) | 0.003 | 5 (50) | 0.060 | 108 (10.6) | <0.001 | 4 (13.8) | 0.270 |
| Ischemic heart disease | 95 (3.4) | 13 (13.7) | 0.099 | 1 (25) | 1.000 | 9 (14.5) | 0.050 | 3 (10.3 | 0.480 |
| Diabetes | 273 (9.8) | 36 (13.2) | 0.013 | 6 (42.9) | 0.110 | 18 (9.6) | 0.306 | 12 (16.9) | 0.010 |
| Renal disease | 22 (0.8) | 5 (22.7) | 0.040 | 1 (50) | 0.420 | 3 (16.7) | 0.160 | 1 (50) | 0.150 |
| Site of index admission (ICU^^^) n (%) | 321 (11.5) | 38 (11.8) | 0.050 | 5 (50) | 0.060 | 78 (6.9) | 0.020 | 86 (7.6) | 1.000 |
| Hemoglobin (g/dL): n (%)  <13.2 for males or <11.6 for females | 587 (21.) | 63 (25.3) | 0.800 | 14 (23.3) | 1.000 | 38 (8.1) | 0.750 | 20 (7.8) | 0.890 |
| Platelet (10*3/uL): n (%) <135 for males or <157 for females | 124 (4.4) | 12 (4.8) | 0.160 | 11 (23.4) | 1.000 | 21 (7.6) | 1.000 | 22 (11.7) | 0.020 |
| White blood cells n (%): >9.2 (10*3/uL) | 952 (34.1) | 93 (37.3) | 0.260 | 21 (24.4) | 1.000 | 40 (7.2) | 0.540 | 33 (10.3) | 0.030 |
| Blood Glucose n (%): >110 mg/dL | 1639 (58.6) | 149 (59.8) | 0.430 | 35 (27.8) | 0.150 | 81 (7.6) | 0.730 | 44 (8.2) | 0.510 |
| Creatinine level n (%): >1.2 mg/dL | 559 (20) | 52 (20.9) | 0.500 | 5 (17.2) | 0.490 | 37 (8.5) | 0.450 | 14 (11.8) | 0.070 |
| Blood urea nitrogen n (%): >20 mg/dL | 622 (22.3) | 58 (23.3) | 0.610 | 13 (28.9) | 0.430 | 36 (8.2) | 0.670 | 12 (7.3) | 1.000 |
| Sodium n (%): <135 mEq/L  >153 mEq/L | 398 (14.2)  15 (0.5) | 47 (18.9)  1 (0.4) | 0.040 | 11 (33.3) | 0.190 | 25 (9)  1 (10) | 0.390 | 11 (12.5) | 0.100 |
| Potassium n (%): <3.5 mmol/L  >5.5 mmol/L | 43 (1.5)  186 (6.7) | 5 (2)  19 (7.6) | 0.380 | 1 (50)  6 (27.3) | 0.610 | 4 (12.1)  10 (8.7) | 0.420 | 3 (6.1) | 0.800 |
| Calcium n (%): <8.4 mg/dL  >10.5 mg/dL | 170 (6.1)  65 (2.3) | 16 (6.4)  7 (2.8) | 0.620 | 1 (14.3)  1 (14.3) | 1.000 | 9 (7.7)  4 (11.8) | 0.630 | 6 (13)  2 (6.9) | 0.260 |
| INR^^^^ n (%): >1.2 | 379 (13.9) | 35 (14.1) | 0.120 | 11 (26.8) | 0.680 | 46 (7.2) | 0.490 | 15 (6) | 0.350 |

^LOS: length of stay; ^^CCI: Charlson Comorbidity Index; ^^^ICU: intensive care unit; ^^^^ INR; international normalized ratio
