# Supplementary material for: Predicting unplanned readmissions in neurological patients: A large-scale AI-driven analysis using CHAID decision trees
Source: PLoS One. 2026 Oct 9;21(10):e0360077. doi: 10.1371/journal.pone.0360077 (PMC13652259; doi:10.1371/journal.pone.0360077)
Supplement: Supplementary file 5 [file pone.0360077.s005.docx]

| **Table S5: Causes of Index Admissions within the sub-Categories of Predictors on CHAID^ Model (N=2795)** | | | | | | |
| --- | --- | --- | --- | --- | --- | --- |
| First predictor | Second predictor | | Third predictor | | Cause of index admission | |
|  |  |  |  |  | Cause | n (%) |
| CCI^^ Score of zero | Governorate | North | No third predictor | | Disc  Back pain  Seizure  Dizziness  Low LOC^^^  High ICP^^^^  Brain hemorrhage  AVM * | 492 (72.5)  11 (1.6)  71 (10.5)  6 (0.9)  23 (3.4)  4 (0.7)  62 (9.1)  10 (1.5) |
|  |  | Middle and south |  |  |  |  |
| CCI Scores 1 till 4 | Age | < 40 years | Gender: | Male | CVA**  Brain hemorrhage  Disc  Brain tumor  others | 28 (16.9)  33 (19.9)  32 (19.3)  62 (37.3)  10 (6) |
|  |  |  |  | female |  |  |
|  |  | 40 to 79 years | LOS*** | <=13 days | CVA  Brain hemorrhage  Disc  Back pain  Brain tumor  Low LOC  Seizure  AVM  dizziness | 506 (32.1)  452 (28.5)  385 (24.3)  16 (1)  112 (7.1)  37 (2.3)  40 (2.5)  8 (0.5)  26 (1.6) |
|  |  |  |  | >13 days |  |  |
|  |  | >=80 years | No third predictor | | Brain hemorrhage  Low LOC  Dizziness  others | 49 (68.1)  11 (15.3)  6 (8.3)  6 (8.4) |
| CCI Scores 5 and 6 | No other predictors | | | | CVA  Brain hemorrhage  Brain tumor  others | 213 (63.8)  74 (22.2)  32 (9.6)  15 (4.5) |
| CCI Score 7 | No other predictors | | | | CVA  Brain hemorrhage  Brain tumor  others | 9 (32.1)  6 (21.4)  10 (35.7)  3 (10.7) |

^CHAID: chi-squared automatic interaction detection, ^^CCI: Charlson Comorbidity Index, ^^^ LOC: Low Level of Consciousness, ^^^^ICP: Intracranial Pressure, *AVM: Arteriovenous Malformation, **CVA: Cerebrovascular Accident, ***LOS: Length of Stay
